# Discovery and identification of candidate genes from the chitinase gene family for *Verticillium dahliae* resistance in cotton

**DOI:** 10.1038/srep29022

**Published:** 2016-06-29

**Authors:** Jun Xu, Xiaoyang Xu, Liangliang Tian, Guilin Wang, Xueying Zhang, Xinyu Wang, Wangzhen Guo

**Affiliations:** 1 State Key Laboratory of Crop Genetics & Germplasm Enhancement, Hybrid Cotton R&D Engineering Research Center, Ministry of Education, Nanjing Agricultural University, Nanjing 210095, China; 2 College of Life Science, Nanjing Agricultural University, Nanjing 210095, China

## Abstract

*Verticillium dahliae*, a destructive and soil-borne fungal pathogen, causes massive losses in cotton yields. However, the resistance mechanism to *V. dahilae* in cotton is still poorly understood. Accumulating evidence indicates that chitinases are crucial hydrolytic enzymes, which attack fungal pathogens by catalyzing the fungal cell wall degradation. As a large gene family, to date, the chitinase genes (*Chis*) have not been systematically analyzed and effectively utilized in cotton. Here, we identified 47, 49, 92, and 116 *Chi*s from four sequenced cotton species, diploid *Gossypium raimondii* (D_5_), *G. arboreum* (A_2_), tetraploid *G. hirsutum* acc. TM-1 (AD_1_), and *G. barbadense* acc. 3–79 (AD_2_), respectively. The orthologous genes were not one-to-one correspondence in the diploid and tetraploid cotton species, implying changes in the number of *Chi*s in different cotton species during the evolution of *Gossypium*. Phylogenetic classification indicated that these *Chis* could be classified into six groups, with distinguishable structural characteristics. The expression patterns of *Chis* indicated their various expressions in different organs and tissues, and in the *V. dahliae* response. Silencing of *Chi23*, *Chi32*, or *Chi47* in cotton significantly impaired the resistance to *V. dahliae*, suggesting these genes might act as positive regulators in disease resistance to *V. dahliae*.

Chitinases (EC 3.2.1.14), which are found in a variety of organisms, catalyze the hydrolysis of the β-1-4-linkage in the N-acetyl-D-glucosamine polymer of chitin, a major component of fungal cell walls and arthropod exoskeletons^1^. Hence, they play a major role in defense by directly attacking invading fungal pathogens. Analysis of the amino acid sequences of these enzymes reveals that chitinases have multiple domains; for example, a typical plant chitinase possesses a signal region at the N-terminal and a catalytic domain, and only vacuolar chitinases have C-terminal regions. In addition, some chitinases also have one or more chitin-binding domains (CBDs) behind the N-terminal signal region, and a variable linking region following the chitin-binding domains^2^,^3^,^4^,^5^.

Of the glycoside hydrolase (GH) gene families, families 18 and 19 are considered as chitinases due to the sequence similarity of their catalytic domains^2^. Based on the amino acid sequence homology, three-dimensional (3D) structures, substrate specificity, mechanisms of catalytic reactions, sensitivity to inhibitors and other characteristics, chitinases have been phylogenetically categorized into five classes (class I-V)^3^,^4^,^5^,^6^,^7^,^8^. Of them, GH family 18 enzymes in classes III and V are more widespread and distributed in a greater diversity of organisms such as fungi, bacteria, viruses, animals, and higher plants than other enzymes. However, members of GH family 19, which contains class I, II, and IV chitinases, are found mainly in higher plants and some bacteria^8^. Structurally, class I chitinases have an N-terminal cysteine-rich CBD and a catalytic domain (CatD) at the C-terminal. These two domains are linked by rich proline and glycine, and the length and composition of the linkers are variable^9^. Class II chitinases have high sequence homology with class I chitinases at the CatD but they lack the CBDs and linker regions. Class III chitinases possess lysozyme activity, and do not have any sequence homology with either class I or II chitinases. However, they do have a high sequence similarity to chitinases in the same class in different plants^10^. Class IV chitinases, which have CBDs and CatDs, are similar to the class I chitinases, but both domains are smaller than those in class I due to one deletion in the CBD and three deletions in the CatD. Class V chitinases possess two CBDs in tandem^11^. The multiple domains and the complex structures in chitinases are indicative of the variety of functions of these enzymes.

On the basis of their physicochemical properties and enzymatic activity, plant chitinases are considered as pathogenesis-related (PR) proteins and have been categorized as PR-3, PR-4, PR-8, and PR-11^4^. In practice, plant chitinases are more propitious to inhibit the growth of fungi by producing hypersensitive reactions and inducing defense responses^12^. These enzymes also generate or degrade some signaling molecules that function by regulating the cell polarity, cell movement and cell division in plant growth and development^13^,^14^. Moreover, the expression patterns or production of the chitinases can be regulated by various abiotic stresses such as wounds, cold, drought, heavy metals, ozone, excessive salinity, and UV light^15^,^16^,^17^,^18^, and a variety of plant phytohormones including jasmonic acid (JA), salicylic acid (SA), ethylene (ET), cytokinin (CK), and indole acetic acid (IAA)^19^.

Cotton is the most important source of natural textile fibers and oilseed in the world, but the production of cotton is constrained by various biotic and abiotic stresses. Among the biotic stresses, fungi are the most severe pathogenic microorganisms. They deliver effector molecules into the plant cell to promote virulence and cause disease^20^. *V. dahliae*, a soil-borne fungal pathogen, is considered as the most serious factor in cotton yield and quality loss^21^,^22^. The most effective method of developing pathogen-resistant varieties is to mine key genes. Members of the chitinase family are pathogenesis-related proteins, and have been identified in almost all plants. Many of these genes have been proven to inhibit fungal growth *in vitro*^23^,^24^, and the overexpression of chitinase genes from some plants or the fungus *Trichoderma* species had shown to enhance resistance to fungal pathogens in a variety of transgenic plants^25^,^26^,^27^,^28^. However, to date, the chitinase genes in cotton have not been systematically analyzed. Although several chitinases from other organisms have been transformed into cotton, the resistance of these transgenic lines to pathogens is not obvious^29^.

The availability of data on the whole-genome of *Gossypium* in different cotton species, such as *G. raimondii* (D5)^30^, *G. arboreum* (A2)^31^, *G. hirsutum* acc. TM-1 (AD1)^32^, and *G. barbadense* acc. 3–79 (AD2)^33^, made it possible to identify chitinase genes on a genome-wide scale, and to enrich our understanding of the molecular mechanism of resistance to *V. dahliae* in cotton. Here, we surveyed chitinase members in four sequenced cotton species, and analyzed their phylogenetic relationships, gene structure, and expression patterns in different tissues and in response to pathogens and hormone treatments. Furthermore, we verified the functional roles of three chitinase genes that are significantly induced in cotton in response to *V. dahliae* by virus-induced gene silencing (VIGS) analysis. Our studies might provide effective gene resources for improving cotton *V. dahliae* resistance in future cotton-breeding programs.

## Results

### Genome-wide identification of chitinase genes and their chromosomal distribution

The whole genome sequence scaffolds of four sequenced cotton species, *G. raimondii*^30^, *G. arboreum*^31^, *G. hirsutum* acc. TM-1^32^, and *G. barbadense* acc. 3–79^33^ were used for the genome-wide exploration of chitinase genes in *Gossypium*. We used the HMMER 3.0 and the Pfam protein family databases with the Glyco_hydro_18 (PF00704) and Glyco_hydro_19 (PF00182) domains to search for chitinases in the four cotton genomes. SMART^34^ and INTERPROSCAN^35^ programs were used to verify the predicted genes. As a result, we identified 47, 49, 92, and 116 *Chi*s in *G. raimondii*, *G. arboreum*, *G. hirsutum* acc. TM-1, and *G. barbadense* acc. 3–79, respectively (Supplementary Table S1). From the phylogenetic view, one member in the diploid *G. raimondii* would correspond to one homologous gene in *G. arboreum* and two homeologs from the A and D subgenomes in tetraploid *G. hirsutum* acc. TM-1 and *G. barbadense* acc. 3–79. Here, 38 members showed such a one-to-one correspondence. Other inconsistencies might result from the different sequencing methods, assembly error in partial chromosomal regions, or duplication in the evolutionary process of *Gossypium*. In particular, in *G. barbadense* acc. 3–79, more tandem *Chis* were found compared with the other three cotton species and need to be further confirmed.

**Figure 1:**
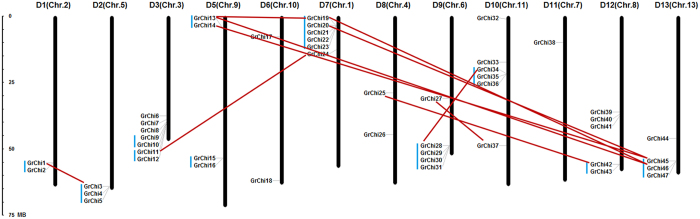
Chromosomal distribution of chitinase genes in *G. raimondii*. The chromosome numbers are displayed at the top of each bar. The 47 chitinase genes in *G. raimondii* are distributed on the linkage map, and the names of the scaffolds from the genome are indicated in brackets. The chromosome numbers from D1 to D3, and D5 to D13, were consistent with the newly-updated interspecific genetic map of allotetraploid cultivated cotton species (Wang *et al*. 2015). The nomenclature of the chitinase genes was based on the order of the chromosomes in *G. raimondii*. The tandem duplication genes are marked in blue lines, and the red lines were drawn to connect segmental duplication genes.

The nomenclature of *Chis* in *Gossypium* followed their chromosome orders. As the *G. raimondii* chromosomes had been integrated with the D-subgenome in *G. hirsutum*^36^, the *Chis* in *G. raimondii* were named preferentially from *GrChi1* to *GrChi47*, based on the reordered *G. raimondii* chromosomes (Fig. 1). The corresponding orthologs in *G. arboreum*, *G. hirsutum* acc. TM-1, and *G. barbadense* acc. 3–79 were named *GaChis*, *GhChis*, and *GbChis*, respectively, with the same number for orthologs as in *G. raimondii* (Supplementary Table S1).

*Chi*s were matched to 12 scaffolds in *G. raimondii* except in the D4 chromosome. In detail, chitinase members were distributed among the *Gossypium* chromosomes unevenly, with D3, D7, and D9 chromosomes observed to have a relatively high density of *Chis* and other chromosomes containing sparse *Chis*. To further ascertain the expansion mechanism of the *Chis* in *G. raimondii*, we analyzed their tandem and segmental duplication events on the 12 chromosomes. Eleven tandem duplications involved in 30 *Chis* were detected in the *G. raimondii* genome, indicating that tandem duplications were the major factor in the expansion of more than 60% of members of the gene family. Moreover, 10 segmental duplication events in 47 *Chis* were identified. The results were quite different from those in *Arabidopsis thaliana* and *Populus trichocarpa*, with 24 *Chi*s found in each chromosome in *A. thaliana*, but 37 *Chi*s were distributed in only eight of 19 chromosomes in *P. trichocarpa*^37^.

### Phylogenetic classification and structural analysis of chitinase genes in *G. raimondii*

**Figure 2:**
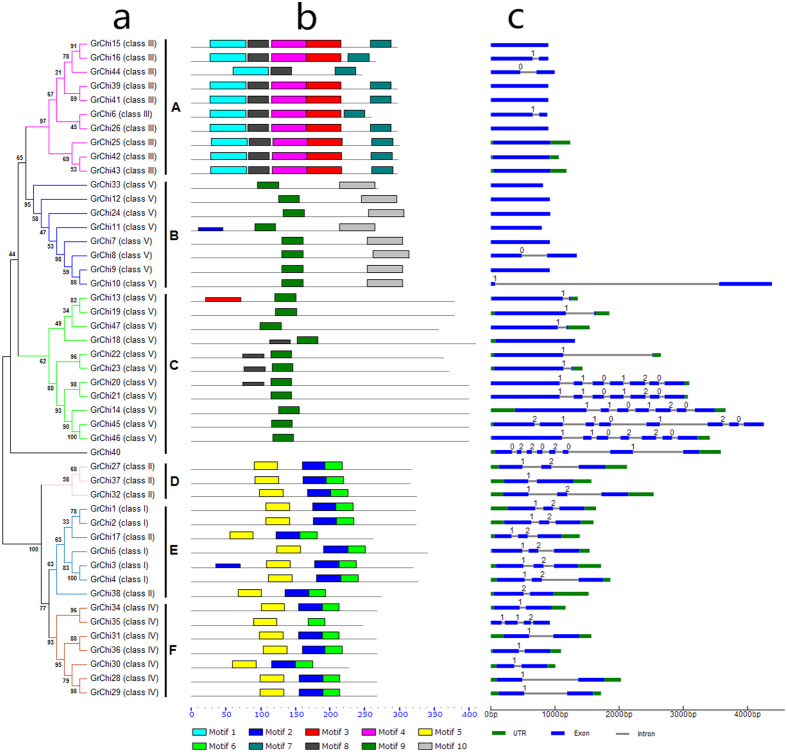
Phylogenetic classification and structural analysis of chitinase genes in *G. raimondii*. (**a**) A phylogenetic tree was made using the maximum likelihood method with 1,000 resampling replicates. The 47 chitinases were clustered into groups A, B, C, D, E, and F; (**b**) Motif locations of chitinases were analyzed, and ten types of motifs were used to detect. The number and order of motifs in each chitinase are shown; (**c**) The gene structure (exon-intron organization) of the chitinase genes in *G. raimondii* are shown, with high accordance with the classification of the chitinase gene family.

With *GrChis* as example, the phylogenetic relationships were further analyzed by constructing an unrooted tree using the maximum likelihood method. The results indicated that the 47 *GrChis* were clustered into six well-supported groups; namely groups A, B, C, D, E, and F (Fig. 2a). These classifications were consistent with the presence of conserved domains and catalytic domains (Fig. 2b), and exon-intron organizations (Fig. 2c).

Thirty chitinases belonging to the GH 18 family were divided into groups A, B, and C, and seventeen members of the GH 19 family were classified into groups D, E, and F. The genes in group A matched those in class III of the previous classifications^3^,^4^,^5^,^6^,^7^,^8^. The chitinase genes of groups B and C corresponded to class V. The class II genes were divided in groups D and E. The genes in class I were classified into group E, and the chitinase genes in group F corresponded to class IV (Fig. 2a).

The motif locations of chitinases are displayed schematically in Fig. 2b. Totally, ten motif components in the 47 *GrChi*s were analyzed. Of them, *GrChi*s in GH 18 gene family were involved in the motifs 1, 3, 4, 7, 8, 9 and 10, and those in GH 19 gene family were possessed of the motifs 2, 5 and 6, respectively. Moreover, we also analyzed the chitin-binding conserved domains in the 47 *GrChi*s, while only eight *GrChi*s belonging to GH 19 gene family was detected, implying that the chitin-binding conserved domain in most *GrChi*s had lost in the evolutionary process. The type, order, number, and motif location of these chitinases were similar within each group, but significantly different to the other groups. In detail, 90% (9 out of 10) of the members in group A displayed the same five motif components (motifs 1, 3, 4, 7, and 8) and were ranked in an identical order, but had many obvious differences to the other five groups. The previously reported class V was further divided into groups B and C in the present study, with many differences in motif locations. Most of the *GrChis* in group B shared motifs 9 and 10, but *GrChi*s in the group C were mainly motif 9. *GrChi*s in groups D, E, and F contained the same three motifs (motifs 2, 5, and 6), which were distributed in the same order. This led to these 3 groups having a closer evolutionary and phylogenetic relationship. These findings indicate that the motif compositions of each group in chitinase gene family are relatively conserved (Supplemental data 1).

To gain further insights into the structural evolution and phylogenetic classification of the genes, the exon-intron organization of *GrChi*s was also analyzed by comparing the corresponding genomic sequences with their coding sequences (CDS) through the software Gene Structure Display Server (GSDS) 2.0 (http://gsds.cbi.pku.edu.cn/) (Fig. 2c). As a result, most genes in the same group generally shared exon-intron organizations with the same number of exons or introns. For example, seven of the genes in group A only had one exon, but the other three genes had two exons. However, the genes of the groups B and C, which were categorized into class V in previous studies, had distinct structural divergences. Most genes in group B only possessed one exon, while more than two exons were found in group C. The majority of genes in groups D and E had three exons, while most genes in group F had two exons.

Overall, the motif locations, motif components, and exon-intron structures of the chitinases in *G. raimondii* supported the phylogenetic classification well. Moreover, the highly conserved sequences of chitinase family members in the same group indicated these genes were subject to duplications during their evolution.

### Expression profiles of chitinase genes in *G. hirsutum* acc. TM-1

**Figure 3:**
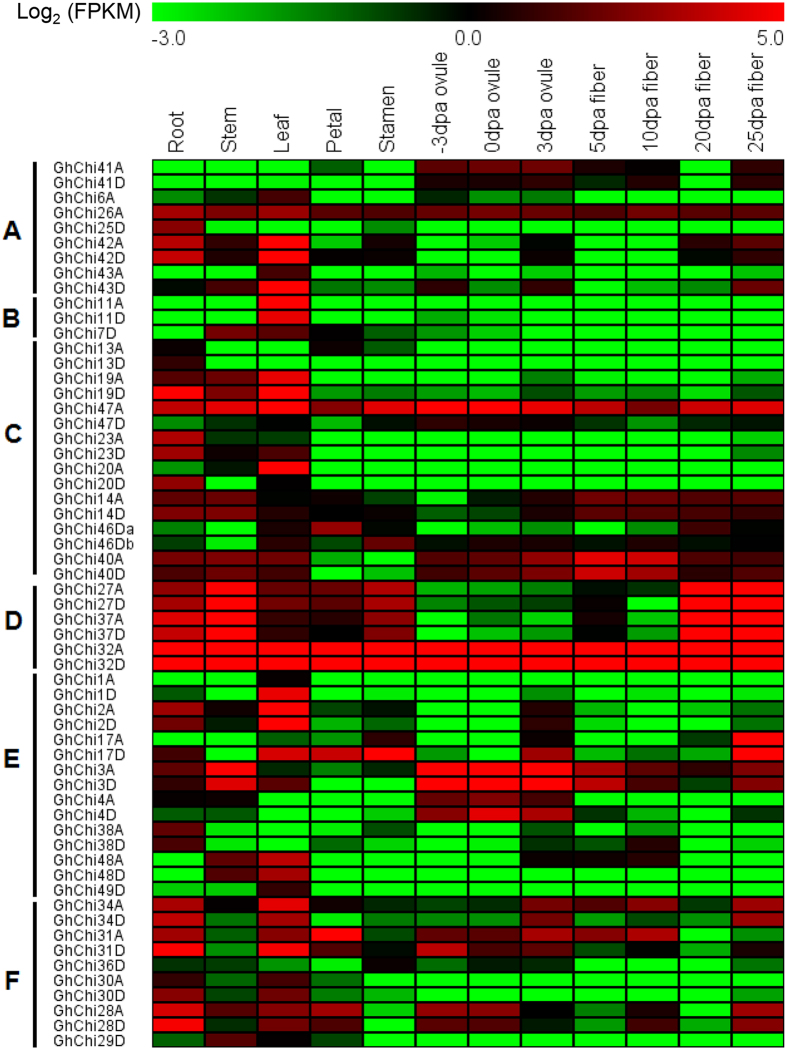
Transcriptional profiling of chitinase genes in different tissues and organs in *G. hirsutum* acc. TM-1. Roots; stems; leaves; petals; stamens; and ovules at −3, 0, and 3 DPA; and fibers at 5, 10, 20, and 25 DPA were used for the comparative transcriptome analysis. The expression data was converted with Log_2_ (FPKM) to calculate *GhChis* expression levels. Gene expression differences are shown in the colors indicated in the scale. The RNA-Seq data used here could be downloaded from http://www.ncbi.nlm.nih.gov/bioproject/PRJNA248163/.

To investigate the roles of chitinase genes in tetraploid cotton, transcriptome data from *G. hirsutum* acc. TM-1 vegetative tissues (root, stem, and leaf), floral tissues (petal and anther), ovule tissues (−3, 0, and 3 DPA), and fiber tissues (10, 20, and 25 DPA) at different developmental stages were used to investigate the gene expression patterns^32^. With FPKM > 1.0, a total of 59 *GhChis* were found to be expressed, and showed diverse developmental and spatial regulation in the various tissues (Fig. 3).

Most of the chitinase genes in group A and group B were predominantly expressed in vegetative organs. *GhChi47A* in group C was ubiquitously expressed in both vegetative and reproductive organs, *GhChi14A*/*D* was undetectable in the ovule, *GhChi40A*/*D* was undetectable in the petal and stamen, and *GhChi46D* was expressed highly in the leaf, petal and the fiber tissues, while other members were only highly expressed in vegetative organs. *GhChi32A*/*D* in group D were expressed at high levels in both vegetative and reproductive organs, yet *GhChi27A*/*D* and *GhChi37A*/*D* were most highly expressed in the stem and fiber tissues. Moreover, eight genes in group E were predominantly expressed in vegetative organs, while *GhChi17A*/*D* had higher expression levels at 25DPA fibers. In addition, *GhChi3A*/*D* and *GhChi4A*/*D* were predominantly expressed in the ovule at different developmental stages. A total of ten genes in group F were detected. Of these, *GhChi28A*/*D, GhChi30D*, *GhChi31A/D*, *GhChi34A*/*D* had high expression levels in roots and leaves. Furthermore, the genes including *GhChi28A*/*D, GhChi31A*/*D*, and *GhChi34A*/*D* were also highly expressed in the reproductive organs. These findings indicate that chitinase genes share diverse but overlapping expression patterns in various tissues and organs, suggesting that chitinase genes have conserved structures but diverse functions.

### Expression analysis of chitinase genes in response to *V. dahlia*

**Figure 4:**
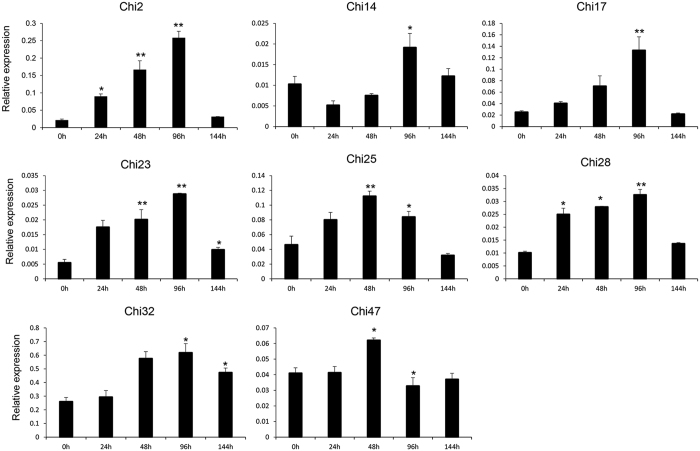
Real-time qRT-PCR analysis of the chitinase genes in response to *Verticillium dahliae* in *G. barbadense* cv. Hai7124. qRT-PCR expression analysis of chitinase genes to screen for differentially expressed genes after inoculation with *V. dahliae* strain V991. The error bars were calculated based on three biological replicates using standard deviation. “*”: significant difference at P < 0.05; “**”: significant difference at P < 0.01.

The expression patterns of chitinase genes have been studied in a range of species, and many genes have been demonstrated to function in resistance to biotic and abiotic stresses^14^,^15^,^16^. To confirm the induction of chitinase genes after *V. dahliae* inoculation, the expression patterns of 23 chitinase genes with detectable expression levels in root tissues following transcriptome analysis were investigated. Of them, eight genes showed high expression levels at various treatment time points, showing that they were significantly induced by *V. dahliae*, and quickly reached a peak at different time points (Fig. 4). In detail, the expression of six genes, *Chi2*, *Chi14*, *Chi17*, *Chi23*, *Chi28*, and *Chi32*, significantly increased after *V. dahliae* inoculation, reaching a peak at 96 h. The other two genes, *Chi25* and *Chi47*, were significantly induced and quickly reached a peak at 48 h after *V. dahliae* treatment. Therefore, the eight genes might be responsible for *V. dahliae* resistance in cotton.

### Functional characterization of candidate genes in cotton responsive to *V. dahliae* through virus-induced gene silencing (VIGS)

By integrating the phylogenetic classification, the expression patterns in various organs and tissues, and in response to *V. dahilae*, the functions of chitinase genes are multiple and complex, with the important roles in disease resistance. To further investigate the function of *Chi*s in *V. dahliae* resistance, we selected three candidate genes, *Chi23*, *Chi32*, and *Chi47*, with different expression levels that were significantly induced after *V. dahliae* inoculation, for functional identification through VIGS analysis.

**Figure 5:**
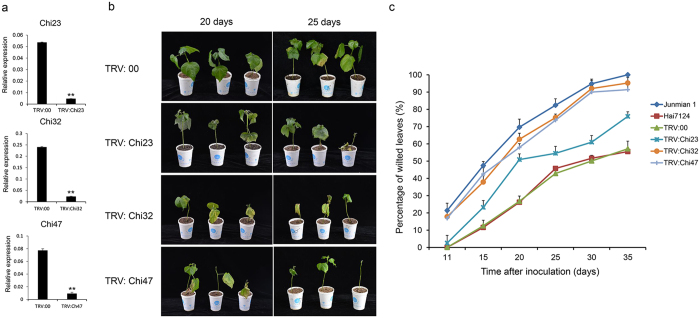
Silencing of *Chi23*, *Chi32*, and *Chi47* significantly impaired the resistance to *V. dahliae* in cotton. Three chitinase genes were first silenced by VIGS in *G. barbadense* cv. Hai7124 seedlings exhibiting *V. dahliae* resistance. The seedlings were then inoculated with *V. dahliae* at a concentration of 1 × 10^7^ spores/mL. (**a**) The transcription levels of *Chi23*, *Chi32*, and *Chi47* in the *Chi23*-silenced, *Chi32*-silenced, *Chi47*-silenced and TRV: 00 plants, and the roots of the VIGS plants were harvested for RNA isolation at two weeks after *Agrobacterium* infiltration; **(b)** Plant phenotypes at 20 and 25 days after *V. dahliae* inoculation; the VIGS plants were dip-inoculated with *V. dahliae* conidia suspension (1 × 10^7^conidia/mL) at two weeks after *Agrobacterium* infiltration; **(c)** The percentage of diseased leaves after *V. dahliae* inoculation. The experiments were repeated using at least 20 seedlings per treatment. The error bars were calculated on the basis of three biological replicates using standard deviation.

We constructed TRV: *Chi23*, TRV: *Chi32*, and TRV: *Chi47* vectors to silence endogenous genes in Hai7124, with TRV: 00 considered as the mock treatment. To validate the viability of VIGS in cotton, CLOROPLASTOS ALTERADOS 1 (*CLA1*), which encodes 1-deoxy-D-xylulose-5- phosphate synthase, was used as an indicator gene and was silenced to produce plants with a bleached leaf phenotype^38^. All constructs were infiltrated into at least 20 Hai7124 seedlings at 8 days post-emergence, and untreated plants were also cultured in the same environment. Two weeks later, all individuals infiltrated with TRV: *CLA1* showed highly uniform bleaching in newly emerged leaves (Fig. S1). Meanwhile, the cotton seedlings confirmed to be infiltrated with all constructs and those with the mock treatment were selected for RNA isolation and quantitative real-time PCR (qPCR) analysis. The untreated and mock-treated plants showed the same or higher expression levels of *Chi23*, *Chi32*, and *Chi47*. However, the transcripts of these three genes showed that they were significantly silenced in plants infiltrated with TRV: *Chi23*, TRV: *Chi32*, and TRV: *Chi47* (P < 0.01) (Fig. 5a).

After the target genes were silenced, we inoculated the cotton seedlings with *V. dahliae* isolate V991 by dip-infection with a final concentration of 1 × 10^7^ spores per milliliter. In parallel, two cotton cultivars, Hai7124 and Junmian 1, were used as controls resistant to and susceptible to *V. dahliae*, respectively, and these were also inoculated with V991. Ten days after inoculation, the seedlings of the Junmian 1 plants showed obvious cotyledon wilting. However, a small number of Hai7124 plants with the leaf wilting phenotype appeared at least 15 days after inoculation with *V. dahliae*. As a result, Hai7124 and Junmian 1 plants were confirmed to be resistant and susceptible to *V. dahliae*, respectively. The phenotype*s* of the two cotton cultivars at 20 and 25 days after inoculation with *V. dahliae* are shown in Fig. S2.

About three weeks after inoculation, the cotton seedlings of gene-silenced plants, particularly those containing TRV: *Chi32* and TRV: *Chi47*, exhibited more wilting and etiolated leaves than the control TRV: 00 seedlings (Fig. 5b). Similarly, all the true leaves in the Junmian 1 seedlings were defoliated 25 days after inoculation (Fig. S2). The defoliated phenotype was also observed in TRV: *Chi32* and TRV: *Chi47* seedlings 30 days after inoculation. In summary, silencing *Chi23, Chi32*, and *Chi47* obviously increased susceptibility to *V. dahliae* in Hai7124 seedlings. In addition, we used at least 20 plants per treatment to calculate the rate of diseased plants. As a result, the TRV: 00 control plants had few wilted leaves, similar to the number seen in Hai7124 seedlings without injection, and the rate of average diseased leaves to healthy leaves was approximately 55% 35 days after inoculation. However, about 95% of the *Chi32*- silenced and *Chi47*-silenced plants showed leaves wilting or exfliation that were similar to the Junmian 1 susceptible control plants, of which 100% were diseased 35 days after inoculation (Supplemental data 2). Furthermore, about 80% of the *Chi23*-silenced plants were diseased: this was higher than the percentage of control plants, but lower than the percentage of *Chi32*-silenced, *Chi47*-silenced, and Junmian 1 plants (Fig. 5c). By comparing the rate of disease in treated and control plants, the silencing of *Chi23*, *Chi32*, and *Chi47* were found to significantly confer the resistance to *V. dahliae* (P < 0.01). These results indicate that *Chi32* and *Chi47* are important genes in resistance to *V. dahliae* infection in cotton, and have potential uses in cotton disease-resistant breeding.

**Figure 6:**
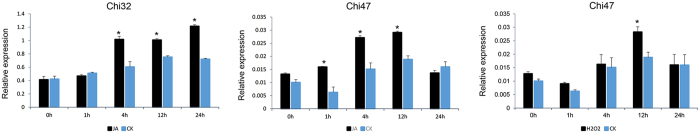
Expression patterns of two chitinase genes treated with stress-related signals in cotton. Two treatments [jasmonic acid (JA) and H_2_O_2_], and only JA were detected the induced changes of the expression levels of *Chi47* and *Chi32*, respectively. “*”: significant difference at P < 0.05.

The expression patterns of chitinase genes are also regulated by various plant phytohormones, including JA, SA, ET, CK, and IAA^19^. To further investigate the molecular mechanisms of *Chi23, Chi32* and *Chi47*, we analyzed the expression of four stress-related signaling compounds, ET, JA, SA, and the hydrogen peroxide (H_2_O_2_). *Chi32* and *Chi47* were found to be induced by one or more of these compounds. The transcript levels of *Chi32* significantly increased and accumulated 4–24 h after JA treatment. *Chi47* was also significantly upregulated and reached a peak 12 h after JA or H_2_O_2_.treatment (Fig. 6). These results imply that *Chi32* and *Chi47* participate in JA and other signaling pathways to protect plants from various stresses.

## Discussion

Chitinases are found in a variety of species including plants, animals, fungi, and bacteria^1^. They not only play a major role in defense against fungal pathogens^16^, but also have an important function in the regulation of growth and development in plants^13^,^14^. As a larger gene family, it is important to elucidate the multiple functions of chitinase genes and to determine their corresponding functional specificity for utilization in genetic analyses and breeding. To date, systematic genome-wide investigation of chitinase genes has been reported in many species; however, knowledge of the chitinase genes in cotton is limited, and their systematic investigation has not been reported.

In *Arabidopsis*, 24 chitinase genes have been found, distributed between all five of the chromosomes^39^. In rice, 37 chitinase genes have been found and these are positioned on all chromosomes except chromosome 7^39^. A model woody plant, *P. trichocarpa*, contains 37 chitinase genes with complete ORFs and these are located on eight of the 19 chromosomes^37^. A total of 26 chitinase genes were found in the diploid banana (AA) genome^40^. Here, we systematically identified 47, 49, 92, and 116 candidate chitinase genes in four sequenced cotton species; the diploid cottons *G. raimondii* and *G. arboreum*, and the tetraploid cottons, *G. hirsutum* acc. TM-1 and *G. barbadense* acc. 3–79, respectively. It is noted that more tandem *Chis* were found in *G. barbadense* acc. 3–79 than the other three cotton species, which might result from the different sequencing methods used, assembly errors in partial chromosomal regions, or evolutionary expansion, and this should be further confirmed. In the diploid cotton *G. raimondii*, the chitinase genes were anchored onto 12 chromosomes and could be divided into six subgroups. Characteristics of the genes, such as signal peptides (SPs), GPI-anchor site, and transmembrane domains, were investigated, and the results indicated multiple complex physiological functions in the defense against various stresses and the enhancement of growth and development in cotton.

In accordance with previous reports, the transcription patterns of plant chitinase genes showed both constitutive and induced expression in the present study^14^,^15^,^16^,^26^. Constitutively expressed chitinases mainly participate in physiological functions such as flower development, symbiotic interactions, seed development, somatic embryogenesis, cell division, and programmed cell death^14^,^41^. However, induced expression of chitinases have been recorded in response to numerous abiotic stresses, such as wounding, drought, salinity, cold, heavy metals, ozone, frost, and UV light^15^,^16^,^17^,^18^, as well as biotic stresses, such as infection with fungi, bacteria, viruses, viroids and nematodes^24^,^25^,^26^,^27^,^28^,^41^. Furthermore, the expression of chitinases is regulated by various phytohormones, such as JA, SA, ET, IAA, and CK^19^.

The variation in the expression patterns of the chitinase genes is related to their functional diversity. For example, a rice chitinase III protein, drought-induced protein 3 (DIP3), was significantly induced by drought, salt, and low temperature, and functions by regulating the plant stress response^42^. In grapevine, the expression levels of *Chi1b* and *CH3* rapidly increased after UV-C exposure, and the chitinase activity rose between 4- to 13-fold in bunchstems^43^. *ScChi*, an acidic class III chitinase in sugarcane, showed different expression levels when treated with H_2_O_2_, methyl jasmonate (MeJA), SA, abscisic acid (ABA), polyethylene glycol (PEG), NaCl, CuCl_2_, and low temperature (4 °C), suggesting that it confers positive responses to biotic and abiotic stresses^44^. In this study, we systematically analyzed the expression patterns of chitinase family genes in cotton. Using the transcriptome data of *G. hirsutum* acc. TM-1 vegetative tissues (root, stem, and leaf), floral tissues (petal and anther), ovule tissues (−3, 0, and 3 DPA), and fiber tissues (10, 20, and 25 DPA) at different developmental stages, a total of 59 *GhChis* were found to have diverse developmental and spatial regulation patterns (Fig. 3). Further, we investigated the expression of 23 *GhChis* with detectable expression in root tissues after *V. dahliae* inoculation. Eight genes were found to have significantly higher expression levels following inoculation and were therefore thought to be induced by *V. dahliae* (Fig. 4). These findings indicate that chitinase genes have diverse functions. It is hoped that these data will help mine key genes in various breeding targets such as *V. dahliae* resistance in cotton.

Chitinases have two different mechanisms for the hydrolysis of chitins: substrate-assisted catalysis and acid catalysis^45^,^46^. Although plants have no chitin in their cell walls, chitinase genes play key roles in multiple physiological processes, including growth and development and non-specific stress responses. Plants produce many forms of chitinases, including vacuolar and apoplastic chitinases, in the natural environment or in response to stresses. The production of chitinases had been shown to relate to some biotic or abiotic factors^15^,^47^. In addition, many chitinase genes sourced from plants and fungi have been successfully over-expressed in other plants to combat stresses. Transgenic tobacco (*Nicotiana tabacum*) plants that overexpress *CHIT33* and *CHIT42* from the mycoparasitic fungus *Trichoderma harzianum*, show broad resistance to biotic stresses (fungal and bacterial pathogens) and abiotic stresses (salinity and heavy metals)^48^. *Cht-2* and *Cht-3*, class-I chitinase genes, are constitutively expressed in japonica rice to enhance resistance to blast (*Magnaporthe grisea*)^26^. Transgenic banana (*Musa acuminata* ‘Gros Michel’) with either of two rice chitinase genes (*rcc2* or *rcg3*) showed enhanced resistance to black leaf streak disease caused by the fungus *Mycosphaerella fijiensis*^27^. A rice chitinase gene (*Cht-2; RCC2*) was introduced into the calli of *Italian ryegrass* (*Lolium multiflorum* Lam.), and dramatically increased its resistance to crown rust disease^49^. A transgenic peanut (*Arachis hypogaea* L.) expressing tobacco chitinase was resistant to leaf spot disease^50^. An endochitinase (*ech42*) was cloned from *T. harzianum* and introduced into apple to increase resistance to apple scab^51^. *Ech42* was also isolated from the biocontrol fungus *Trichoderma virens* and successfully expressed in tomato and tobacco to enhance their tolerance to fungal pathogens^28^. In addition, the transient co-expression of class IV chitinase in pepper (*CaChitIV*) and pepper receptor-like cytoplasmic protein kinase (*CaPIK1*) in pepper plants (*Capsicum annuum*) enhanced the *CaPIK1*-triggered cell death response, the abundance of reactive oxygen species (ROS), and the number of nitric oxide (NO) bursts. In contrast, co-silencing of both *CaChitIV* and *CaPIK1* reduced induction of the cell death response, ROS and NO bursts, and defense response genes, and hence, increased susceptibility to *Xanthomonas campestris* pv. *vesicatoria* (*Xcv*) infection^52^. Co-expression of a rice basic chitinase gene (*RCH10*) and a modified maize ribosome-inactivating protein (*MOD1*) in transgenic rice significantly enhanced resistance to sheath blight^25^. Interestingly, transgenic plants expressing chitinase genes in combination with other PR proteins, such as a modified maize ribosome-inactivating protein or β-1, 3-glucanases, showed enhanced resistance to fungal pathogens compared to those where a single chitinase gene was introduced^25^. Together, these results suggest that chitinase genes play important roles in growth, development, and non-specific stress responses, and play a particular role in the defense against fungal pathogens.

In this study, based on genome-wide identification, phylogenetic classification, and expression patterns, we further validated the roles of several chitinase genes in cotton *V. dahliae* resistance. We silenced the *Chi23*, *Chi32*, and *Chi47* genes in *G. barbadense* cv. Hai7124 and found through VIGS analysis that it led to *Verticillium* resistance. Two weeks after infiltration with TRV: *Chi23*, TRV: *Chi32*, and TRV: *Chi47*, seedlings exhibited obvious silencing compared with the controls, and the silencing of these genes significantly enhanced cotton susceptibility to *V. dahliae* (Fig. 5). Statistical analysis suggested that silencing *Chi23*, *Chi32*, or *Chi47* increased the susceptibility of Hai7124 to *V. dahliae*. Interestingly, the *Chi32*-silenced and *Chi47*-silenced plants showed particularly serious *Verticillium* wilt that was similar to that seen in the susceptible plant, Junmian 1. These findings imply that *Chi32* and *Chi47* are important defense-related genes and play crucial roles in resistance to *V. dahliae* in cotton. These systematic analyses of the chitinase family in cotton have provided crucial information on the molecular mechanisms of disease resistance, and provide a solid foundation for disease-resistant cotton breeding.

From the evolutionary view, *G. hirsutum* and *G. barbadense* probably originated from a single hybridization event between A- and D- diploid species, however, the two have very different agronomic and fiber quality characteristics^53^. Modern *G. hirsutum* cultivars have high-yield properties and dominate more than 90% of worldwide cotton production, while *G. barbadense*, characterized by its elite fiber qualities and resistance to *V. dahliae*, accounts for less than 10%^32^,^33^. Even the high similarity of orthologous genes in the two cotton species, the differences involved in the single nucleotide polymorphisms (SNP), expression level, proteins structures, and the variance in epigenetics significantly affected their phenotype characteristics^54^,^55^. Here, we found that *Chi23*, *Chi32* and *Chi47* were significantly upregulated at different expression levels in cotton roots after inoculation with *V. dahlia*, with higher expression in *G. barbadense* cv. Hai7124 than *G. hirsutum* cv. Junmian 1 (Fig. S3). Further function analysis showed that silencing of these three genes in Hai7124, especially *Chi32* or *Chi47*, significantly increased the susceptibility of the cotton to *V. dahliae*. Taken together, our results suggested that resistant genes could be effectively mined from resistant materials, and these *Chi*s might act as positive regulators in disease resistance to *V. dahliae*. Their function in disease resistance will be further elucidated by transgenic expression analysis in susceptible cotton cultivars.

The chitinase genes exhibit multiple complex functions in development and stress responses, and seem to be mainly involved in active or passive defense against pathogens. However, the production of chitinases is also regulated by the generation or degradation of signal molecules such as JA, ET, SA, CK, H_2_O_2_, and auxin^19^. In the bark and sapwood of *Norway spruce* (*Picea abies* L.), the expression levels of a class IV chitinase gene (*PaChi4*) was obviously increased when treated with the necrotropic pathogen *Heterobasidion parviporum* and MeJA^56^. In tomato (*Solanum lycopersicon* L.), *SlERF1*, an ethylene response factor (ERF), was engineered for *Rhizopus nigricans* resistance by accumulating chitinase (*CHI*) and phenylalanine ammonia lyase (PAL)^57^. In *Casuarina equisetifolia*, *t*he expression of *CeChi1* were induced by pathogen infection or SA treatment^58^. *Ltchi7*, a class III chitinase, was significantly induced in roots of *Lotus tenuis* and *L. japonicas* when treated with salt, drought, H_2_O_2_, and ABA^59^. Among these endogenous signaling molecules, the SA-mediated defense responses were effective against hemi-biotrophs and biotrophs, and were critical for systemic acquired resistance, whereas JA tended to act with ET to induce resistance to necrotrophic pathogens^60^.

Many signaling molecules such as the JA, ET, SA, and H_2_O_2_ positively contributed to the disease resistance to *V. dahliae* in cotton^38^,^61^. Accumulating evidence indicated that *V. dahliae*-responsive genes such as *GbSSI2*^38^*, GhSSN*^62^*, GbERF*^63^, and *GbWARKY1*^64^, which were involved in regulating the biosynthesis of signaling molecules, played crucial roles in defense against *Verticillium* wilt. In this study, the expression of *Chi23*, *Chi32* and *Chi47* was further investigated following treatment with the stress-related signals ET, JA, SA, and H_2_O_2_. As a result, the expression levels of *Chi32* and *Chi47* were significantly increased after JA treatment, and the expression of *Chi47* was also upregulated following H_2_O_2_ treatment. Expression analysis showed that *Chi32* and *Chi47* may be involved in resistance to stress through regulation of JA or H_2_O_2_. This, along with VIGS analysis, suggests that JA acts as a key factor in engineering the chitinase gene-mediated resistance to *V. dahliae* in cotton; however the detailed functional mechanisms remains to be investigated.

## Materials and Methods

### Identification of the chitinase gene family in four sequenced cotton species

The available genomic database of four sequenced cotton species, *G. raimondii*, *G. arboreum*, *G. hirsutum* acc. TM-1, and *G. barbadense* acc. 3–79, were downloaded from http://www.phytozome.net/, http://cgp.genomics.org.cn, http://mascotton.njau.edu.cn/, and http://cotton.cropdb.org/cotton/download/data.php, respectively. We used the Pfam protein family databases with the Glyco_hydro_18 (PF00704) and Glyco_hydro_19 (PF00182)^65^ domains and the HMMER software version 3.0^66^ to identify the chitinase genes in the four cotton species. Further, the chitinase genes were isolated from the four databases using SeqHunter software version 1.0. In addition, the sequences were further confirmed the existence of the conserved domains of Glyco_hydro_18 or Glyco_hydro_19 using SMART^34^ and INTERPROSCAN^35^.

### Mapping, phylogenetic tree construction, and structural analysis

The distribution of chitinase genes in *Gossypium* was determined by MapInspect (http://www.plantbreeding.wur.nl/UK/software_mapinspect.html), where the chromosome orders from D1 to D13 and A1 to A13 referred to the map published by Wang *et al*.^36^. *G. raimondii* chromosomes were integrated with the D-subgenome in *G. hirsutum*^36^ and reordered from D1 to D13. The nomenclature of chitinase genes (*GrChis*) in *G. raimondii* was based on the order of the chromosomes. The corresponding orthologs in *G. arboreum*, *G. hirsutum* acc. TM-1, and *G. barbadense* acc. 3–79 were named *GaChis*, *GhChis*, and *GbChis*, respectively, following their orthologs in *G. raimondii*.

Multiple sequence alignments of chitinase proteins were carried out using ClustalX (ver.1.83). A phylogenetic tree was built by MEGA 5.1 software (www.megasoftware.net) using the maximum likelihood method. The exon/intron structures were analyzed by aligning the genomic DNA sequences with their corresponding coding sequences using the online Gene Structure Display Server (GSDS) program (http://gsds1.cbi.pku.edu.cn/). Motifs of chitinase proteins were investigated statistically using MEME (http://meme-suite.org/), which set the maximum number of motifs at 10.

### Plant materials and treatments

Seeds of *G. barbadense* cv. Hai7124, *G. hirsutum* cv. Junmian 1, and *G. hirsutum* cv. Jinmian 19 were used for expression analysis under different stress treatments. They were grown in the different controlled environment chambers under the same conditions of a 16 h light/8 h dark cycle at 28 °C for 2 weeks.

The seedlings of Hai7124 and Junmian 1, which exhibited resistance and susceptibility to *V. dahliae*, respectively, were inoculated with the fungal pathogen (*V. dahliae*) with the dip-inoculation method^67^. V991, a highly aggressive defoliating strain of *V. dahliae*, was cultured on potato dextrose agar medium at 24 °C for 4–5 d, and then incubated in Czapek’s medium (NaNO_3_, 0.3% w/v; MgSO_4_, 0.1% w/v; KH_2_PO_4_, 0.1% w/v; FeSO_4_, 0.0002% w/v; KCl, 0.1% w/v; Sucrose, 3% w/v; pH6.0) at 25 °C for 5 d. We then adjusted the concentration to 10^7^ conidia per ml using deionized water for inoculation of the seedlings^38^. The seedling roots were harvested with three repeats at different time points (0, 24, 48, 96, and 144 hours) after V991 treatment, and then quick-frozen in liquid nitrogen and stored at −80 °C for use.

*G. hirsutum* cv. Jinmian 19, which exhibits high tolerance to abiotic stress, was used for the stress signal treatments. The leaves of three-week-old cotton seedlings were sprayed with 100 μM JA, 1 mM ET, 100 mM SA, or 10 mM H_2_O_2_ (ddH_2_O as a solvent control), and the leaves were collected at different time points (0, 1, 4, 12, and 24 hours). Three repeats were harvested at each time point and stored at −80 °C after quick-freezing in liquid nitrogen.

### RNA isolation and expression pattern analysis

Total RNA was extracted from cotton seedling leaves or roots using the CTAB-acidic phenol extraction method^68^. RNA was then treated with DNase I (Invitrogen, http://www.invitrogen.com/) to remove genomic DNA, and 2 μg of total RNA was used for first-strand cDNA synthesis using the Superscript first-strand synthesis system (Invitrogen, Foster City, CA).

The primer pairs used for qRT- PCR analysis were designed by Beacon Designer 7.0 according to the chitinase gene sequences in *G. raimondii* (Supplementary Table S2). The amplified fragment lengths were set between 80 base pair (bp) and 200 bp, and the annealing temperature was controlled at 60 °C. The cotton histone3 (AF024716) gene was used as the reference gene for qRT- PCR.

The real-time PCR amplification reactions were performed on an ABI 7500 Real Time PCR System (Applied Biosystems, USA) using SYBR Green (Vazyme, Nanjing, China) with three replicates. The amplification parameters were: denaturation at 95 °C for 10 min, 40 cycles of denaturation at 95 °C for 15 s, annealing at 60 °C for 15 s, and extension at 72 °C for 15 s. Expression data from three biologically independent experiments were analyzed and presented as means ± S.D.

To analyze the expression of chitinase genes, we obtained *G. hirsutum* acc. TM-1 high-throughput RNA-sequencing data from Zhang *et al*.^32^ from vegetative tissues (root, stem, and leaf), floral tissues (petal and stamen), ovule, and fiber tissues at −3, 0, 3, 10, 20, and 25 days post anthesis (DPA), respectively. Finally, Log_2_ (FPKM) was used to calculate *GhChis* expression levels, where FPKM referred to fragments per kilobase of exon model per million mapped reads with Cufflinks software (http://cufflinks.cbcb.umd.edu/).

### Cloning of the chitinase genes in *G. barbadense* cv. Hai7124

Based on the known sequences of four cotton species, we designed gene-specific primers to amplify the homologous genes of chitinases with complete open reading frames (ORFs) in *G. barbadense* cv. Hai7124, a species resistant to *V. dahliae* (Supplementary Table S2). Standard PCR reactions were performed using High-fidelity ExTaq DNA Polymerase (TaKaRa Biotechnology [Dalian] Co. Ltd., China). All the PCR products were cloned into pMD19-T cloning Vector (TaKaRa, Dalian, China) and transformed into *E. coli* DH5α. At least six clones per gene were randomly selected and sequenced.

### Vector construction and functional characterization of candidate genes via virus-induced gene silencing (VIGS) analysis

The pTRV1 and pTRV vectors for VIGS analysis were generously provided by Dr. Libo Shan of Texas A & M University (College Station, TX, USA). pTRV: *GhCLA1*, where GhCLA1 (Cloroplastos alterados 1) encodes 1-deoxy-D-xylulose-5-phosphate synthase, was used as a control^69^. The TRV: *Chi23* construct contained a 444-bp fragment of the *Chi23* cDNA, the TRV: *Chi32* construct included a 454-bp fragment of *Chi32*, and the TRV: *Chi47* construct included a 387-bp fragment of *Chi47*. These fragments were cloned from Hai7124 by PCR using primers with *Eco*RI/*Sac*I enzyme sites for TRV: *Chi23* and TRV: *Chi32*, and *Eco*RI/*Bam*HI enzyme sites for TRV: *Chi47*, for insertion into TRV. The primer pairs used for constructing the VIGS vectors are displayed in Table S2.

We transformed the vectors, TRV1, TRV: 00, TRV: *GhCLA1*, TRV: *Chi23*, TRV: *Chi32*, and TRV: *Chi47* into *Agrobacterium tumefaciens* strain GV3101, and cultured these *Agrobacterium* at 28 °C in LB medium containing the antibiotics 50 μg/mL kanamycin and 25 μg/mL rifampicin for 2 days. We then cultured the *Agrobacterium* in liquid LB medium (50 μg/mL kanamycin, 25 μg/mL rifampicin) on a shaking table with 160 rpm per minute at 28 °C for 16 hours. Finally, we inoculated the *Agrobacterium* at a concentration of 1:100 into the liquid LB medium that contained the antibiotics rifampicin (25 μg/mL) and kanamycin (50 μg/ mL), shook the mixture, and allowed the *Agrobacterium* to grow to an OD_600_ of 0.5 at 28 °C. The *Agrobacterium* was pelleted using centrifugation at 4000 rpm at room temperature for 10 min, and the OD_600_ value was adjusted to 2.0 with infiltration media (10 mM MgCl_2_, 10 mM 2-Morpholinoethanesulfonic acid, and 200 μM acetosyringone).

The cell suspensions carrying TRV1, TRV: 00, TRV: *Chi23*, TRV: *Chi32*, and TRV: *Chi47* were incubated at room temperature for 3 h, and then infiltrated into the eight-day-old Hai7124 cotton seedlings with two fully expanded cotyledons using a needleless 1 mL syringe at a 1:1 ratio. For mock treatment and the technical control, the same plants were infiltrated with a 1:1 mixture of *Agrobacterium* carrying TRV1 and TRV: 00 or TRV1 and TRV: *CLA1*, respectively. All the plants were grown in the same growth chamber at 23/21 °C (day/night), with a 16 h light/8 h dark cycle. About 8 days after *Agrobacterium* infiltration, the seedlings infiltrating with TRV: *CLA1* showed a bleached leaf phenotype. Two weeks after *Agrobacterium* infiltration, the TRV: *CLA1* plants exhibited highly uniform bleaching in newly emerged leaves, and then we selected three seedlings per treatment and harvested the leaves of the VIGS plants for the RNA isolation. Meanwhile, the cotton seedlings containing the susceptible control Junmian 1, the resistant control Hai7124, and all the VIGS plants were removed from the soil and dip-inoculated with *V. dahliae* conidia suspension (1 × 10^7^conidia/mL) as previously described^67^. All plants were grown in the same growth chamber at 25/23 °C (day/night), with a 16 h light/8 h dark cycle for seven weeks to investigate the rate of the diseased leaves to healthy leaves. The VIGS experiments were repeated at least three times and each treatment was applied to more than 20 plants to increase the reliability of the results.

## Additional Information

**How to cite this article**: Xu, J. *et al*. Discovery and identification of candidate genes from the chitinase gene family for *Verticillium dahliae* resistance in cotton. *Sci. Rep*. **6**, 29022; doi: 10.1038/srep29022 (2016).

## Supplementary Material

Supplementary Information

Supplementary Table S1

Supplementary Table S2
