# Supplementary material for: Discovery and identification of candidate genes from the chitinase gene family for Verticillium dahliae resistance in cotton
Source: Sci Rep. 2016 Jun 29;6:29022. doi: 10.1038/srep29022 (PMC4926273; doi:10.1038/srep29022)
Supplement: Supplementary Information [file srep29022-s1.pdf]

Discovery and identification of candidate genes from the chitinase gene family for *Verticillium dahliae* resistance in cotton

Jun Xu[1], Xiaoyang Xu[1], Liangliang Tian[1], Guilin Wang[1], Xueying Zhang[1], Xinyu Wang[2]*, Wangzhen Guo[1]*

[1]State Key Laboratory of Crop Genetics & Germplasm Enhancement, Hybrid Cotton R & D Engineering Research Center, Ministry of Education, Nanjing Agricultural University, Nanjing 210095, China

[2]College of Life Science, Nanjing Agricultural University, Nanjing 210095, China

*** Correspondence and requests for materials should be addressed to W.Z.G or X.Y.W. (email: moelab@njau.edu.cn; xywang@njau.edu.cn)**

Supporting Information

Table S1 Identification of chitinase genes in *G. raimondii* and their phylogenetic relationship in *G. arboreum*, *G. hirsutum* and *G. barbadense*.

Table S2 Information of PCR primers used in this study.

Figure S1. Silencing of the endogenous Cloroplastos alterados gene (*GbCLA1*) in cotton through VIGS. The leaf bleaching phenotype was observed two weeks after infiltration in TRV: *GbCLA1* plants.

Figure S2. Infection of cotton seedlings with *V. dahliae*. Two cotton cultivars, *G. barbadense* cv. Hai7124 and *G. hirsutum* cv. Junmian 1, were used as controls that were resistant and susceptible to *V. dahliae*, respectively. The seedlings were grown in the same environment and inoculated with V991. We identified the phenotype 20d and 25d after inoculation. In Junmian 1, all the true leaves were defoliated 25 days after inoculation.

Figure S3. Real-time qRT-PCR analysis of the chitinase genes in response to *Verticillium dahlia* in *G. hirsutum* cv. Junmian 1. qRT-PCR expression analysis of chitinase genes to screen for differentially expressed genes after inoculation with *V. dahliae* strain V991. The error bars were calculated based on three biological replicates using standard deviation. “*”: significant difference at P < 0.05.

Table S1 Identification of chitinase genes in *G. raimondii* and their phylogenetic relationship in *G. arboreum*, *G. hirsutum* and *G. barbadense*

**Gene name**	**Gene ID in *Gossypium raimondii***	**Gene name**	**Gene ID in D subgenome of *Gossypium hirsutum***	**Gene name**	**Gene ID in A subgenome of *Gossypium hirsutum***	**Gene name**	**Gene ID in *Gossypium arboreum***	**Gene name**	**Gene ID in D subgenome of *Gossypium barbadense***	**Gene name**	**Gene ID in A subgenome of *Gossypium barbadense***
GrChi1	Gorai.002G203500.1	GhChi1D	Gh_D01G1682	GhChi1A	Gh_A01G1442	GaChi1	Cotton_A_36866	GbChi1D	Gbscaffold3179.10	GbChi1A	Gbscaffold3179.9
GrChi2	Gorai.002G203600.1	GhChi2D	Gh_D01G1683	GhChi2A	Gh_A01G1443	GaChi2	Cotton_A_36865	GbChi2D1	Gbscaffold11871.2	GbChi2A	Gbscaffold3179.8
								GbChi2D2	Gbscaffold11871.3		
GrChi3	Gorai.005G257900.1	GhChi3D	Gh_D02G2284	GhChi3A	Gh_A03G1844	GaChi3	Cotton_A_00601	GbChi3D	Gbscaffold17185.6	GbChi3A	Gbscaffold10683.16
GrChi4	Gorai.005G258000.1	GhChi4D	Gh_D02G2285	GhChi4A	Gh_A03G1845	GaChi4	Cotton_A_00600	GbChi4D1	Gbscaffold17185.7	GbChi4A	Gbscaffold10683.14
						GaChi5d	Cotton_A_00599	GbChi4D2	Gbscaffold17185.8		
						GaChi5c	Cotton_A_00598	GbChi4D3	Gbscaffold10683.15		
				GhChi5bA	Gh_A03G1846	GaChi5b	Cotton_A_00597			GbChi5A1	Gbscaffold10683.12
GrChi5	Gorai.005G258100.1	GhChi5D	Gh_D02G2286	GhChi5aA	Gh_A03G1847	GaChi5a	Cotton_A_00596			GbChi5A2	Gbscaffold10683.13
GrChi6	Gorai.003G123100.1	GhChi6D	Gh_D03G1110	GhChi6A	Gh_A03G0434	GaChi6	Cotton_A_20307	GbChi6D1	Gbscaffold4557.6	GbChi6A	Gbscaffold4557.7
								GbChi6D2	Gbscaffold14947.7		
								GbChi6D3	Gbscaffold4557.5		
GrChi7	Gorai.003G129600.1	GhChi7D	Gh_D03G1178	GhChi7A	Gh_A03G2083	GaChi7	Cotton_A_30644	GbChi7D	Gbscaffold14202.5	GbChi7A	Gbscaffold1415.3
GrChi8	Gorai.003G134100.1	GhChi8D	Gh_D03G1228	GhChi8A	Gh_A03G0349	GaChi8	Cotton_A_18542			GbChi8A1	Gbscaffold1351.2
										GbChi8A2	Gbscaffold11698.6
GrChi9	Gorai.003G135700.1							GbChi9D	Gbscaffold8357.19		
GrChi10	Gorai.003G135800.1	GhChi10D	Gh_D03G1242	GhChi10A	Gh_A03G0338	GaChi10	Cotton_A_37547	GbChi10D	Gbscaffold8357.20		
GrChi11	Gorai.003G138400.1	GhChi11D	Gh_D03G1271	GhChi11A	Gh_A03G0305	GaChi11	Cotton_A_02215	GbChi11D	Gbscaffold4553.4		
GrChi12	Gorai.003G138500.1	GhChi12D	Gh_D03G1272	GhChi12A	Gh_A03G0304	GaChi12	Cotton_A_02214	GbChi12D	Gbscaffold4553.2	GbChi12A	Gbscaffold4553.3
GrChi13	Gorai.009G053800.1	GhChi13D	Gh_D05G0520	GhChi13A	Gh_A05G0404			GbChi13D	Gbscaffold23722.14	GbChi13A	Gbscaffold23722.13

GrChi14	Gorai.009G053900.1	GhChi14D	Gh_D05G0521	GhChi14A	Gh_A05G0405	GaChi14	Cotton_A_01265	GbChi14D	Gbscaffold390.15	GbChi14A	Gbscaffold23722.15
GrChi15	Gorai.009G389400.1	GhChi15D	Gh_D05G3437	GhChi15A	Gh_A04G0259			GbChi15D	Gbscaffold8045.2	GbChi15A1	Gbscaffold15636.3
										GbChi15A2	Gbscaffold8045.1
										GbChi15A3	Gbscaffold15636.2
GrChi16	Gorai.009G389700.1	GhChi16D	Gh_D05G3440	GhChi16A	Gh_A04G1304	GaChi16	Cotton_A_20387	GbChi16D	Gbscaffold28012.1	GbChi16A	Gbscaffold15636.4
GrChi17	Gorai.010G058900.1	GhChi17D	Gh_D06G0479	GhChi17A	Gh_A06G0439	GaChi17	Cotton_A_35181			GbChi17A	Gbscaffold12679.14
GrChi18	Gorai.010G243800.1	GhChi18D	Gh_D13G1952	GhChi18A	Gh_A13G1591	GaChi18	Cotton_A_09095	GbChi18D	Gbscaffold9898.1	GbChi18A	Gbscaffold16912.1
GrChi19	Gorai.001G030000.1	GhChi19D	Gh_D07G0246	GhChi19A	Gh_A07G0192	GaChi19	Cotton_A_07229	GbChi19D	Gbscaffold17255.4	GbChi19A	Gbscaffold9904.8
GrChi20	Gorai.001G030100.1	GhChi20D	Gh_D07G0247	GhChi20A	Gh_A07G0193	GaChi20	Cotton_A_07230	GbChi20D	Gbscaffold17255.5		
GrChi21	Gorai.001G030200.1										
GrChi22	Gorai.001G030300.1	GhChi22D	Gh_D07G0248	GhChi22A	Gh_A07G0194	GaChi22	Cotton_A_07231	GbChi22D	Gbscaffold17255.6	GbChi22A	Gbscaffold50562.2
GrChi23	Gorai.001G030400.1	GhChi23D	Gh_D07G0249	GhChi23A	Gh_A07G0195	GaChi23	Cotton_A_07232	GbChi23D	Gbscaffold17255.7	GbChi23A	Gbscaffold50562.3
GrChi24	Gorai.001G106000.1	GhChi24D	Gh_D07G0925	GhChi24A	Gh_A07G0853	GaChi24	Cotton_A_24671	GbChi24D1	Gbscaffold2211.3	GbChi24A	Gbscaffold4798.21
								GbChi24D2	Gbscaffold2211.2		
GrChi25	Gorai.004G116500.1	GhChi25D	Gh_D08G1052	GhChi25A	Gh_A08G0872	GaChi25	Cotton_A_33972	GbChi25D	Gbscaffold6583.1	GbChi25A	Gbscaffold8325.1
GrChi26	Gorai.004G154400.1	GhChi26D	Gh_D08G1418	GhChi26A	Gh_A08G1134	GaChi26	Cotton_A_22925	GbChi26D1	Gbscaffold9612.14	GbChi26A	Gbscaffold13335.3
								GbChi26D2	Gbscaffold1503.24		
								GbChi26D3	Gbscaffold1503.26		
								GbChi26D4	Gbscaffold13335.5		
GrChi27	Gorai.006G078900.1	GhChi27D	Gh_D09G0628	GhChi27A	Gh_A09G0630	GaChi27	Cotton_A_16545	GbChi27D1	Gbscaffold4505.6	GbChi27A	Gbscaffold7561.3
								GbChi27D2	Gbscaffold7561.4		
GrChi28	Gorai.006G230300.1	GhChi28D	Gh_D09G2016	GhChi28A	Gh_A09G2323	GaChi28	Cotton_A_19782	GbChi28D	Gbscaffold9972.14	GbChi28A1	Gbscaffold9972.13
										GbChi28A2	Gbscaffold9120.17
										GbChi28A3	Gbscaffold9120.18
GrChi29	Gorai.006G230400.1	GhChi29D	Gh_D09G2017	GhChi29A	Gh_A09G2322	GaChi29	Cotton_A_19781	GbChi29D1	Gbscaffold9120.19	GbChi29A	Gbscaffold9972.16
								GbChi29D2	Gbscaffold9120.20		

								GbChi29D3	Gbscaffold9972.15		
GrChi30	Gorai.006G230600.1	GhChi30D	Gh_D09G2019	GhChi30A	Gh_A09G2319	GaChi30	Cotton_A_19779	GbChi30D1	Gbscaffold9120.22	GbChi30A	Gbscaffold9972.18
								GbChi30D2	Gbscaffold9120.24		
GrChi31	Gorai.006G230700.1	GhChi31D	Gh_D09G2020	GhChi31A	Gh_A09G2318	GaChi31	Cotton_A_19778	GbChi31D	Gbscaffold9120.23	GbChi31A1	Gbscaffold9972.19
										GbChi31A2	Gbscaffold9120.25
GrChi32	Gorai.011G005100.1	GhChi32D	Gh_D10G0046	GhChi32A	Gh_A10G0043	GaChi32	Cotton_A_10234	GbChi32D	Gbscaffold18847.8	GbChi32A	Gbscaffold1187.6
GrChi33	Gorai.011G120000.1	GhChi33D	Gh_D10G1066	GhChi33A	Gh_A10G1387	GaChi33	Cotton_A_36274	GbChi33D	Gbscaffold20077.3	GbChi33A	Gbscaffold18684.3
GrChi34	Gorai.011G137200.1	GhChi34D	Gh_D10G1214	GhChi34A	Gh_A10G1271	GaChi34	Cotton_A_16373	GbChi34D	Gbscaffold19845.2	GbChi34A	Gbscaffold19845.1
GrChi35	Gorai.011G137300.1							GbChi35D	Gbscaffold8816.1	GbChi35A1	Gbscaffold6967.1
										GbChi35A2	Gbscaffold2259.20
										GbChi35A3	Gbscaffold2259.21
GrChi36	Gorai.011G137500.1	GhChi36D	Gh_D10G1216	GhChi36A	Gh_A10G1269	GaChi36	Cotton_A_16368			GbChi36A	Gbscaffold2259.17
GrChi37	Gorai.011G198500.1	GhChi37D	Gh_D10G1769	GhChi37A	Gh_A10G1518	GaChi37	Cotton_A_34647	GbChi37D	Gbscaffold4231.19	GbChi37A	Gbscaffold2341.2
GrChi38	Gorai.007G117600.1	GhChi38D	Gh_D11G1103	GhChi38A	Gh_A11G0955	GaChi38	Cotton_A_15696	GbChi38D1	Gbscaffold12094.8	GbChi38A1	Gbscaffold3970.12
								GbChi38D2	Gbscaffold3970.13	GbChi38A2	Gbscaffold12094.9
GrChi39	Gorai.008G118600.1	GhChi39D	Gh_D12G1062	GhChi39aA	Gh_A12G0953	GaChi39a	Cotton_A_31995	GbChi39D	Gbscaffold16250.5	GbChi39A1	Gbscaffold14734.2
						GaChi39b	Cotton_A_31998			GbChi39A2	Gbscaffold16250.6
GrChi40	Gorai.008G124300.1	GhChi40D	Gh_D12G1121	GhChi40A	Gh_A12G1002	GaChi40	Cotton_A_28340	GbChi40D	Gbscaffold4581.4	GbChi40A1	Gbscaffold26044.2
										GbChi40A2	Gbscaffold4581.5
GrChi41	Gorai.008G128500.1	GhChi41D	Gh_D12G1158	GhChi41A	Gh_A12G1039	GaChi41	Cotton_A_25893	GbChi41D	Gbscaffold4.13	GbChi41A	Gbscaffold3794.13
GrChi42	Gorai.008G272000.1	GhChi42D	Gh_D12G2669	GhChi42A	Gh_A12G2714	GaChi42	Cotton_A_17761	GbChi42D1	Gbscaffold6258.18	GbChi42A	Gbscaffold6258.19
								GbChi42D2	Gbscaffold14106.27_scaffold14106.28		
								GbChi42D3			
GrChi43	Gorai.008G272100.1	GhChi43D	Gh_D12G2670	GhChi43A	Gh_A12G2715	GaChi43	Cotton_A_17762	GbChi43D1	Gbscaffold6258.20	GbChi43A	Gbscaffold14106.29
								GbChi43D2	Gbscaffold14106.30		
GrChi44	Gorai.013G168100.1	GhChi44D	Gh_D13G1538	GhChi44A	Gh_A13G1238	GaChi44	Cotton_A_16800	GbChi44D	Gbscaffold14031.8	GbChi44A	Gbscaffold6731.1

GrChi45	Gorai.013G217500.1	GhChi45D	Gh_D13G1990	GhChi45bA	Gh_A13G1628	GaChi45b	Cotton_A_09058	GbChi45D	Gbscaffold4545.4	GbChi45A	Gbscaffold6997.1
						GaChi45a	Cotton_A_09057				
GrChi46	Gorai.013G217600.1	GhChi46Da	Gh_D13G1991			GaChi46	Cotton_A_09056	GbChi46D1	Gbscaffold6881.3	GbChi46A	Gbscaffold9694.1
		GhChi46Db	Gh_D13G1992					GbChi46D2	Gbscaffold6881.4		
GrChi47	Gorai.013G217700.1	GhChi47D	Gh_D13G1993	GhChi47A	Gh_A13G1629	GaChi47	Cotton_A_09055	GbChi47D1	Gbscaffold6881.7	GbChi47A1	Gbscaffold6881.5
								GbChi47D2	Gbscaffold6881.6	GbChi47A2	Gbscaffold9694.2
		GhChi48D	Gh_D11G3475	GhChi48A	Gh_A11G1569	GaChi48	Cotton_A_22147				
		GhChi49D	Gh_D11G1653	GhChi49A		GaChi49	Cotton_A_12561			GbChi49A	Gbscaffold16331.3

Table S2 Information of PCR primers used in this study

Primer name	Description	Primer sequence (5′ → 3′)	Restriction enzyme site (Underlined)
Chi2	qRT-PCR	F: AATGTGGTAAGGGTTGGAAT	
		R: TGGTTGTAGCAGTCAAGATT	
Chi3	qRT-PCR	F: GGTGACTGTATTGACTGCTA	
		R: CATTAATATTCCGAGGGTGTAAA	
Chi13	qRT-PCR	F: GGAGATAGAGGAGTTCAATA	
		R: CGCTGTTATATCATCATCAT	
Chi14	qRT-PCR	F: AATCCAGAGAATAGACCAACCAT	
		R: GTGATGCAGACGTACTTCCT	
Chi17	qRT-PCR	F: AGAGATACTGTGACATACTG	
		R: ATGATACGATGGATTAAAGTG	
Chi19	qRT-PCR	F: GGATTCGTGGCTATTTCTTT	
		R: GAACTACTACTACTGCTACTTG	
Chi23	qRT-PCR	F: GATGATGGTTGGACTCTT	
		R: TTCGTGACTTGTAATAGTATAAC	
Chi25	qRT-PCR	F: AGTATGGAGGTGTTATGCT	
		R: GAGGTTGCTTGCTTCATAT	
Chi26	qRT-PCR	F: TCAAAGGTTCCATCAAGTA	
		R: TAGCACGGTTACAAGATTA	
Chi27	qRT-PCR	F: TTGACACTATGAACAACATC	
		R: GAAGAAGGAGATACAGTGAA	
Chi28	qRT-PCR	F: GGACTGTTGATGATTCTAA	

		R: GATATTGTGTGTTGGTTTC	
Chi29	qRT-PCR	F: GACTAAACTCGCCTGAAAC	
		R: CCTTGGCTTATCACTGGTT	
Chi30	qRT-PCR	F: CTATTGAATGCGATGGTGGGA	
		R: AAGTAGCCGAAACAAGAAAGTA	
Chi31	qRT-PCR	F: TTAATGGAGACCTTGAATG	
		R: AAATGGAATAAACTCTAGCA	
Chi32	qRT-PCR	F: CAACCAAGAATGACACAGAG	
		R: AAGGTAATGGGAGATGATGA	
Chi34	qRT-PCR	F: GCACAAGACAAAGTTCAGC	
		R: AGCATCAGTAGCCTTCAAG	
Chi36	qRT-PCR	F: CCACATAGAAGAGACAGATG	
		R: TAATTGTAGTTCCATGATAGTTG	
Chi37	qRT-PCR	F: CATTACCTTTATTACCTTGAC	
		R: AGAGTTTGTTTCCTTTATTC	
Chi40	qRT-PCR	F: ACTTATGACCTATGACTTCT	
		R: AATCTCTGAGCATTGTTAC	
Chi42	qRT-PCR	F: ACCTAATGTGTTGACTTCT	
		R: AACGGACTTACTATAACCAT	
Chi43	qRT-PCR	F: GCAAGTATGGTGGTGTTATG	
		R: GAGAACGAGAAGTAGCAAGA	
Chi46	qRT-PCR	F: AATAACAAGATACGAAAGGAAGGA	
		R: TGCATAAGCTGTCAGTCATC	
Chi47	qRT-PCR	F: AAGGTTGAGTATGCTAAGAC	
		R: ATTCGTTCCCATGTATTTGA	
Chi23	VIGS Vector construct	F: CGGAATTCTAGCCTGGACTGGATCAACGT	EcoRI

		R: CGAGCTCGCGAAGTAACCAAGCAACCCT	SacI
Chi32	VIGS Vector construct	F: CGGAATTCGCTTCCATTTATGAGCCTTTG	EcoRI
		R: CGAGCTCCTGGTTTCCAGTTACCCACGA	SacI
Chi47	VIGS Vector construct	F: CGGAATTCAAGTGCTGCTGTTTATTTCGC	EcoRI
		R: CGGGATCCTGGGCATAGTGTTCATCATTG	BamHI
Chi23	ORF amplification	F: ATGGCTGCAAAACTCCTTAG	
		R: CTACGATCCCCAAGTTTCCG	
Chi32	ORF amplification	F: TTCTTCAGCTCTAAGCGACAA	
		R: CCACCTAACAGCAGCAAATC	
Chi47	ORF amplification	F: ATGGCTAACGTACTAAGCTC	
		R: CTAGTGCATTCGTTCCCATG	
Chi23	RT-PCR	F: CGGAATTCTAGCCTGGACTGGATCAACGT	
		R: CGAGCTCGCGAAGTAACCAAGCAACCCT	
Chi32	RT-PCR	F: CGGAATTCGCTTCCATTTATGAGCCTTTG	
		R: CGAGCTCCTGGTTTCCAGTTACCCACGA	
Chi47	RT-PCR	F: CGGAATTCAAGTGCTGCTGTTTATTTCGC	
		R: CGGGATCCTGGGCATAGTGTTCATCATTG	

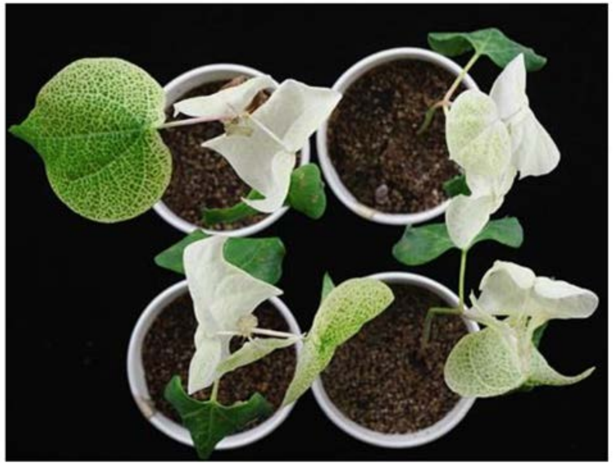

Figure S1. Silencing of the endogenous Cloroplastos alterados gene (*GbCLA1*) in cotton through VIGS. The leaf bleaching phenotype was observed two weeks after infiltration in TRV: *GbCLA1* plants.

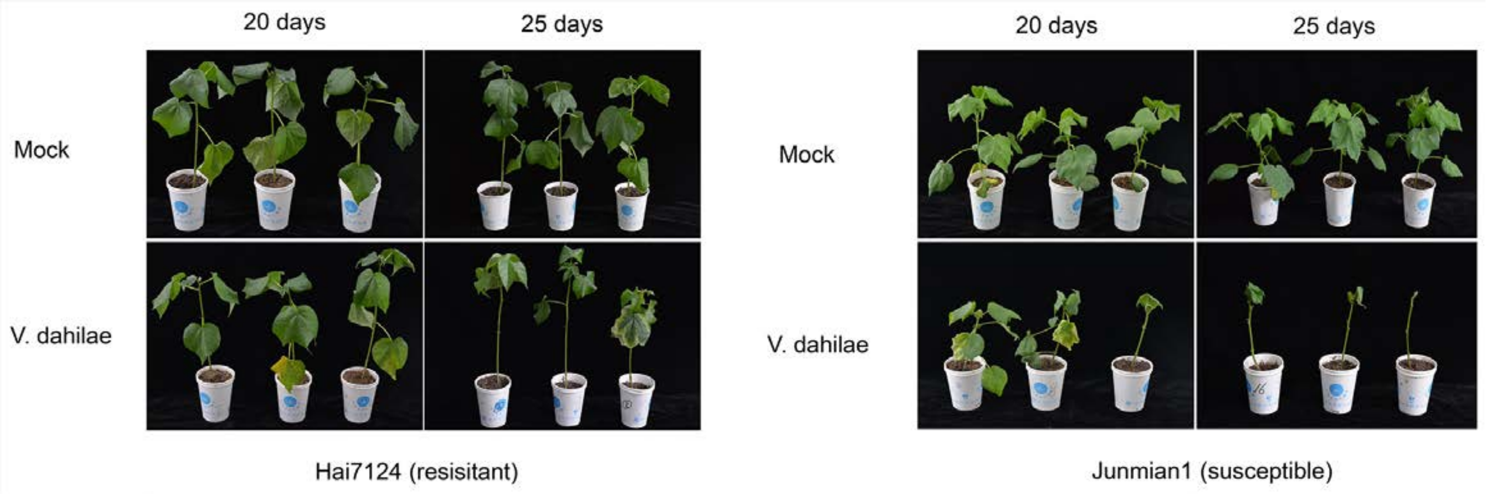

Figure S2. Infection of cotton seedlings with *V. dahliae*. Two cotton cultivars, *G. barbadense* cv. Hai7124 and *G. hirsutum* cv. Junmian 1, were used as controls that were resistant and susceptible to *V. dahliae*, respectively. The seedlings were grown in the same environment and inoculated with V991. We identified the phenotype 20d and 25d after inoculation. In Junmian 1, all the true leaves were defoliated 25 days after inoculation.

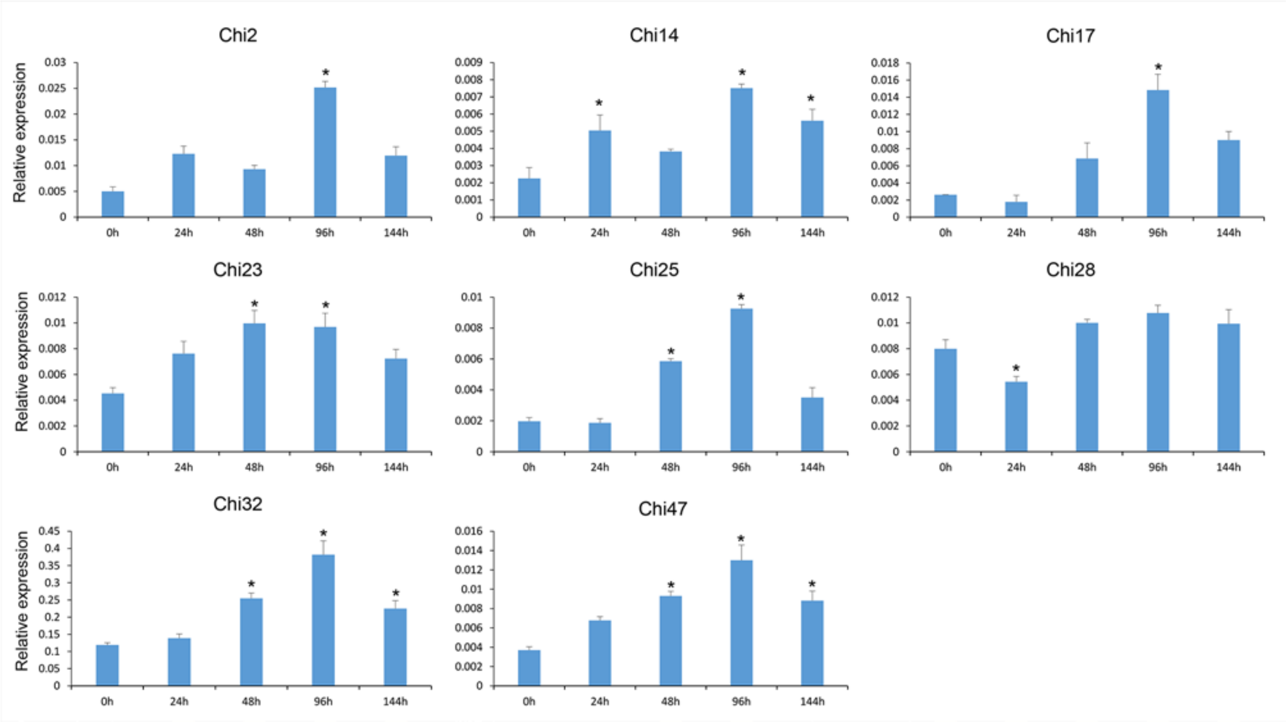

Figure S3. Real-time qRT-PCR analysis of the chitinase genes in response to *Verticillium dahlia* in *G. hirsutum* cv. Junmian 1. qRT-PCR expression analysis of chitinase genes to screen for differentially expressed genes after inoculation with *V. dahliae* strain V991. The error bars were calculated based on three biological replicates using standard deviation. "*": significant difference at P < 0.05.
